# Effect of Bilateral Superficial Cervical Plexus Block on Postoperative Analgesic Consumption in Patients Undergoing Thyroid Surgery

**DOI:** 10.7759/cureus.21212

**Published:** 2022-01-13

**Authors:** Mine Ozgun, Tulay Hosten, Mine Solak

**Affiliations:** 1 Anesthesiology, Yalova State Hospital, Yalova, TUR; 2 Anesthesiology and Reanimation, Faculty of Medicine, Kocaeli University, Kocaeli, TUR

**Keywords:** landmark techniques, cervical plexus, postoperative pain, thyroid surgery, bilateral superficial cervical plexus block

## Abstract

Purpose

Patients complain of moderate-intensity pain following thyroid surgery. Superficial cervical plexus block (SCPB) can be employed as a component of multimodal analgesia after thyroid surgery. This double-blind, randomized study aimed to compare the effects of bilateral SCPB (BSCPB) on postoperative analgesic requirements following thyroid surgery.

Methods

A total of 60 American Society of Anesthesiologists (ASA) I-II patients who underwent elective total thyroidectomy under general anesthesia were randomly assigned to Group 1 and Group 2. After inducing general anesthesia, BSCPB was not administered to Group 1, whereas BSCPB was administered using a three-point injection technique with 0.5% levobupivacaine in Group 2. Patient-controlled analgesia (PCA) was applied by using tramadol in both groups for postoperative analgesia. Tenoxicam was administered as rescue analgesic to patients in case of numeric rating scale (NRS) >4. The postoperative consumption of tramadol, rescue analgesic requirement, and hoarseness, hematoma, signs of local anesthetic toxicity were recorded.

Results

The consumption of tramadol for PCA at two, six, 12, and 24 hours postoperatively, NRS scores in the recovery room, and the number of patients who used tenoxicam as rescue analgesic were significantly lower in Group 2 than in Group 1. The hemodynamic values were similar between the groups.

Conclusions

Our study demonstrates that BSCPB, when applied as a component of multimodal analgesia, is an effective method for reducing the analgesic requirements following thyroid surgery.

## Introduction

Patients complain of moderate-intensity pain following thyroid surgery. However, in the first 24 hours after surgery, some patients require opioid analgesics [1]. Nonsteroidal anti-inflammatory drugs (NSAIDs) can be used for postoperative analgesia as it is relatively well-tolerated and does not have any known side effects of opiates. However, NSAIDs have been reported to increase the risk of postoperative bleeding. Todd et al. and Horlocker suggested that the modern concept of postoperative analgesia includes regional anesthesia techniques [2,3]. Regional nerve blockade has been reported to decrease mechanical hyperalgesia caused by inflammation [4,5]. Superficial cervical plexus block (SCPB) can be employed as a component of multimodal analgesia following thyroid surgery. The superficial cervical plexus has its origins from the ventral rami of the nerve roots C2 to C4. Two nerve loops, which are formed by the union of the adjacent anterior spinal nerves from C2 to C4, give off four superficial sensory branches, listed in cranio-caudal order as follows: lesser occipital (C2, C3), great auricular (C2, C3), transverse cervical (C2, C3), and supraclavicular nerves (C3, C4) [6,7]. These nerve roots provide sensation to the skin, and superficial structures of the ear auricle, acromioclavicular joint, clavicle and anterolateral neck [8]. SCPB consists of a bilateral injection with local anesthetic behind the lateral border of the sternocleidomastoid muscle, which induces surface anaesthesia to the neck. A two- or three-point injection technique can be adopted. Unlike the two-point technique, the three-point technique supplies blockade of the transverse cervical branches of the plexus. The complications of SCPB include hoarseness, hematoma, and local anesthetic toxicity.

Woldegerima et al. concluded that bilateral SCPB (BSCPB) has significantly reduced pain scores, opioid, and total analgesic consumption following thyroid surgery [9]. Similarly Gürkan et al., in their study, concluded that ultrasound-guided BSCPB has a significant analgesic effect in patients undergoing thyroid surgery [10]. 

This double-blind, randomized study aimed to compare the effects of BSCPB on postoperative analgesic requirements following thyroid surgery.

## Materials and methods

A total of 60 patients aged between 18 and 65 years, classified as American Society of Anesthesiologists (ASA) I-II, and scheduled for elective total thyroidectomy under general anesthesia were included after ethics approval was obtained from the local ethics committee of Kocaeli University (approval 3/11). The study was explained to the patients, and written informed consent was obtained from them.* *Data analysis was conducted using the Statistical Package for Social Sciences (SPSS) 13.0 statistical software system (SPSS Inc., Chicago, IL, USA).* *Continuous variables with normal distribution were expressed as mean ± standard deviation, and the categorical variables were expressed as percentages or numbers. The continuous variables were analyzed using the Mann-Whitney U test and, the categorical variables using the Chi-squared test. A *P *value of <0.05 was considered statistically significant.

The exclusion criteria were hypothyroidism, hyperthyroidism, premedication with preoperative analgesics, excessive alcohol intake, electrolyte imbalance, retrosternal goiter, pregnancy, coagulation disorders, and additional surgical interventions. Before the operation, all the patients were informed of the postoperative pain scale (numeric rating scale (NRS), 0 = no pain, 10 = worst imaginable pain) and intravenous patient-controlled analgesia (PCA). In addition, the patients were randomized using the sealed envelope technique. After premedication with midazolam 0.03 mg kgˉ 1 IV, the patients were transferred to the operating room, and their noninvasive blood pressure, peripheral oxygen saturation and heart rate (HR) were monitored. After preoxygenation, anesthesia was induced using pentothal 5-7 mg kgˉ1, fentanyl 1 µg kgˉ1, and rocuronium 0.6 mg kgˉ1, all administered intravenously. General anesthesia was maintained with 4-L dkˉ1 sevoflurane in a mixture of oxygen and nitrous oxide. The bispectral index (BIS; Aspect Medical Systems, Newton, MA, USA) was monitored, and sevoflurane was adjusted to maintain a BIS score between 40 and 60. After giving proper surgical position to neck, BCSPB was not administered to Group 1 (n = 30), whereas in Group 2 (n =30), it was performed with 10 mL for each side (right and left), total 20 mL of 0.5% levobupivacaine using the three-point injection technique and by the same anesthesiologist (MO). The head of the patient was slightly rotated toward the contralateral side that will be anesthetized, and 6 mL of local anesthetic was injected subcutaneously from 2 cm below the mastoid process to 2 cm above the clavicle along the posterior border of the sternocleidomastoid muscle after an aspiration test using a 21-gauge needle at each 2 cm. Subsequently, the needle was inserted into the midpoint of the sternocleidomastoid muscle, and 3 mL of local anesthetic was injected horizontally above the muscle to block the transverse cervical nerve. Finally, 1 mL of local anesthetic was injected subcutaneously to the point of puncture to block the supraclavicular nerves. The injection depth was not >5 mm so as to prevent the block of the phrenic or recurrent laryngeal nerve. Additional doses of fentanyl (50 µg) were administered for variations of systolic blood pressure (SBP) and HR of more than 20% when compared with the control values (before the induction of anesthesia-t0) in two groups. The SBP, HR, and minimum alveolar concentration of sevoflurane (MACsev) were recorded postinduction (t1), postintubation (t2), during incision (t3), 10 min after incision (t4), 30 min after incision (t5), 90 min after incision (t6), and 120 min after incision (t7). Twenty minutes before the end of the surgery, the patients were intravenously administered with tramadol 1 mgkgˉ 1 bolus and were taken to the recovery room (RR) after extubation. The NRS scores for pain were evaluated upon admission to the RR, and at one, two, six, 12, and 24 hours postoperatively, PCA was initiated by using tramadol in both groups for postoperative analgesia in the RR; tramadol consumption at one, two, six, 12, and 24 hours postoperatively was recorded. In addition, tenoxicam was intravenously administered for rescue analgesic in case of NRS >4. NRS >6 indicates acute pain, and the number of patients who had a pain score ≥6 at any time during the first 24 hours following surgery was recorded. The patients and the anesthesiologist responsible for the follow-up of the patients during the postoperative period were blinded to the group allocation. Block complications such as hoarseness, hematoma, and signs of local anesthetic toxicity that occurred during the postoperative period were recorded. The primary outcome was the effect on postoperative analgesic consumption, and the secondary outcome was the comparison of its effect on intraoperative analgesic and anesthetic consumption with postoperative complications.

## Results

The demographic and anesthetic characteristics of the two groups were similar. No significant difference was observed in the fentanyl requirements between the groups during anesthesia (Table 1). The consumption of tramadol was significantly lower in Group 2 than in Group 1 at all times, except for the first hour after surgery (Table 2). In addition, the NRS scores were lower upon admission to the RR in Group 2 than Group 1, in other assessment times were similar (Table 3 ).

**Table 1:** Demographic, anesthetic and surgical characteristics (mean ± SS, n)

	Group 1 (n = 30)	Group 2 (n = 30)	P
Age, yr	48.17 ± 14.3	43.97 ± 0.8	0.177
Gender, female/male (n)	22/8	26/4	0.197
Height, cm	163.4 ± 9.6	160.9 ± 6.2	0.818
Weight, kg	73.03 ± 13.9	74.03 ± 12.0	0.640
Duration of surgery, min	115.40 ± 19.5	118.33 ± 20.5	0.585
Number of patients who required fentanyl, (n)/%	4/13	3/10	0.690
MACsev during anesthesia	1.16 ± 0.12	1.15 ± 0.16	0.482

**Table 2:** Comparison of tramadol consumption by patient-controlled analgesia (PCA) device between the groups (mean ± SD)

Assessment time	Group 1	Group 2	P
1^st^ h.	48.0 ± 28.1	40.6 ± 31.7	0.31
2^nd^ h.	95.3 ± 48.6	66.3 ± 39.8	0.02*
6^th^ h.	163.0 ± 76.6	108.8 ± 58.9	0.004*
12^th^ h.	222.4 ± 95.9	147.6 ± 83.6	0.002*
24^th^ h.	260.6 ± 108.5	164.3 ± 96.3	0.001*

**Table 3:** Postoperative numeric rating scale (NRS) scores between the groups (mean ± SD)

Assessment time	Group 1	Group 2	P
At RR	3.3 ± 2.2	2.0 ± 1.6	0.013*
1^st^ h.	3.0 ± 1.8	2.7 ± 1.8	0.400
2^nd^ h.	2.6 ± 1.7	2.5 ± 1.7	0.752
6^th^ h.	2.2 ± 1.8	2.0 ± 1.2	0.752
12^th^ h.	1.4 ± 1.1	1.5 ± 1.3	0.807
24^th^ h.	0.6 ± 0.7	0.5 ± 0.9	0.363

**Figure 1:**
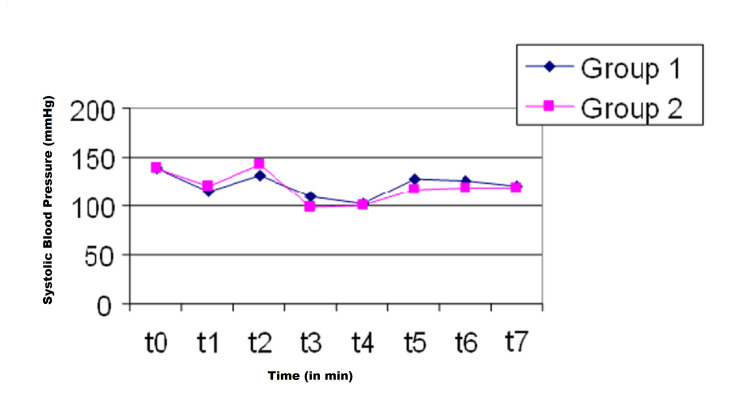
Systolic blood pressure (SBP) during anesthesia

**Figure 2:**
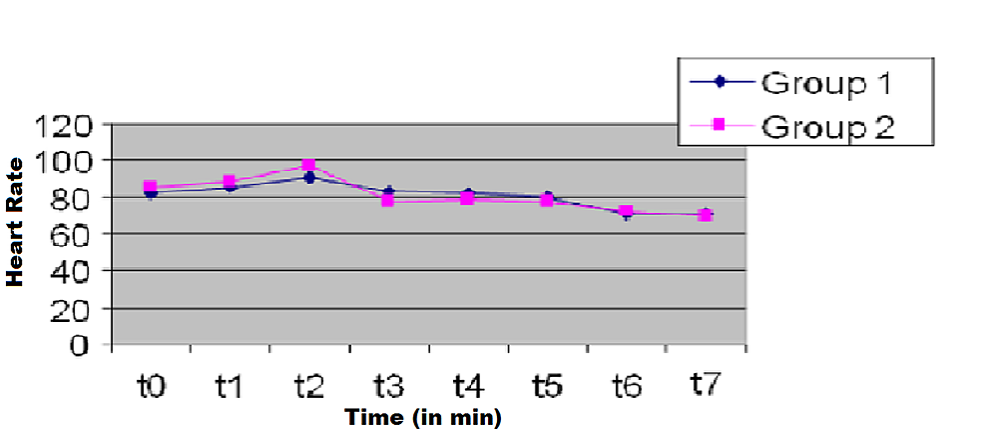
Heart rate (HR) changes during anesthesia

Similar hemodynamic responses were obtained during surgery (Figures 1, 2). No significant difference was observed in the mean MACsev between the groups during anesthesia (Table 1). The mean MACsev was lower at t3 and t4 in Group 2 than in Group 1. The number of patients requiring rescue analgesic was significantly higher in Group 1 (11, 36%) than in Group 2 (4, 13%) (P = 0.03). The number of patients who had a pain score of ≥6 was significantly lower in Group 2 (3, 10%) than in Group 1 (10, 33%) (P = 0.02). 

Postoperative subcutaneous emphysema was detected around the neck in two patients in Group 2. Emphysema regressed at the end of the 12th hour. There were no other complications related to BSCPB that occurred in the patients.

## Discussion

In this study, we demonstrated that BSCPB, which was performed immediately after the induction of general anesthesia in patients undergoing thyroid surgery using a three-point technique with 0.5% levobupivacaine, significantly reduced postoperative analgesic consumption, rescue analgesic consumption requirement and pain severity. However we could not demonstrate any difference in the intraoperative opioid requirements and anesthetic agent consumption. The NRS score upon admission to the RR was significantly lower in Group 2 than in Group 1, but we could not demonstrate any difference in the other postoperative NRS scores. In addition, no significant difference was observed in the mean MACsev between the groups during anesthesia. The significant drop in the use of anesthetics at the t3 and t4 times in the block-induced group was not considered to be clinically significant. 

There are different results of the studies on BSCPB for post-thyroidectomy pain. Dieudonne et al. employed a three-point injection and demonstrated that BSCBP (20 mL of 0.25% bupivacaine) decreased the postoperative pain intensity and postoperative opioid requirement [11]. Furthermore, Andrieu et al. [12] reported that BSCPB with ropivacaine (0.487%) or ropivacaine and clonidine was effective in reducing analgesic requirements following thyroid surgery. On the contrary, Herbland et al. suggested that BSCPB (0.75% ropivacaine) before or after surgery did not improve postoperative analgesia following total thyroidectomy [13]. Eti et al. (30 mL of 0.25% bupivacaine or 20 mL 0.25% bupivacaine with local wound infiltration) reported that neither local wound infiltration nor BSCPB decreased the opioid requirement or pain scores following thyroid surgery [14]. Herbland et al. [13] and Eti et al. [14] performed BSCPB without any adjuvants, such as epinephrine in the study by Dieudonne [11] or clonidine in the study by Andrieu et al. [12]. Herbland et al. performed BSCPB using the two-injection technique [13]. However, with the three-injection technique additional infiltration of the transverse cervical branches can be achieved. The advantage of the three-point injection is the additional analgesia in the aforementioned area. In addition to the aforementioned factors (type of local anesthetic, volume and concentration of local anesthetic, block technique, and addition of an adjuvant agent), different postoperative analgesia protocols (PCA, intermittent bolus), and postoperative analgesic agents (opioids, NSAIDs), pain evaluation intervals, and different pain scoring scales (NSR, VAS) may lead to different BSPCB results in terms of postoperative analgesic efficacy.

Woldegerima et al. reported that BSCPB using 10 mL of 0.25% bupivacaine just before induction prolonged the time to first analgesic requirement, and reduced opioid and total analgesic consumption in the first 24 hours postoperatively [9]. Karakis et al. demonstrated a reduction in opioid consumption and postoperative pain intensity in patients who underwent thyroid surgery [15].

In our study, it can also be concluded that BSCPB reduced the severity of pain, since the number of patients with NRS scores higher than 6 in Group 2 was significantly lower than that in Group 1.

BSCPB can be performed before or after operation for pain control. Mayhew et al. [16] demonstrated that performing BSCPB before the operation reduces the postoperative analgesic requirement compared with performing it after the operation.

Messner et al. reported that SCPB is safe, easy to perform, and effective in reducing morphine consumption, and improving pain relief following carotid endarterectomy under general anesthesia [17]. We employed the three-point injection technique by the anatomical landmarks (LMs). The two- and three-point techniques are LM methods for BSCPB. Ultrasound (US) is another method for nerve localization for BSCPB. It allows direct visualization of the nerves and needle movement. US-guided BSCPB decreases the complication rates [10,18]. Senapathi et al. [19] compared the effectiveness of US-guided versus LM techniques for BSCPB in thyroidectomy. US-guided BSCPB was more effective in reducing pain both intra- and postoperatively compared with the LM technique.

It has been reported that bilateral deep cervical plexus block and combined bilateral superficial and deep cervical plexus block reduced not only postoperative but also intraoperative analgesic requirements [20]. However, serious deep block complications, especially phrenic nerve paralysis, limit the bilateral application of this block [21].

Previous studies did not mentioned subcutaneous emphysema as a complication of BSCPB, but in our study postoperatively subcutaneous emphysema was observed around the neck in two patients in Group 2. Emphysema regressed at the end of the 12th hour. No other complications related to BSCPB occurred in the patients.

BSCPB can reduce postoperative analgesic requirements but is insufficient for pain following post-thyroidectomy. In addition, it can be easily and safely performed; thus, this technique can be used as a component of multimodal analgesia.

## Conclusions

The present randomized study demonstrates that performing BSCPB using the three-point injection technique with 0.5% levobupivacaine is effective in reducing systemic analgesic requirements following thyroid surgery. BSCPB performed using the landmark technique has a very low risk of complications; it is also safe and can be performed in a short time. As a component of the multimodal analgesia approach, BSCPB can be employed for thyroid surgery.
